# Neuroprotective Effect of Ultrasound Triggered Astaxanthin Release Nanoparticles on Early Brain Injury After Subarachnoid Hemorrhage

**DOI:** 10.3389/fchem.2021.775274

**Published:** 2021-10-21

**Authors:** Wei Cai, Qi Wu, Zhi Zhong Yan, Wei-Zhen He, Xiao-Ming Zhou, Long-Jiang Zhou, Jian-Yong Zhang, Xin Zhang

**Affiliations:** ^1^ Department of Neurosurgery, Jinling Hospital, the First School of Clinical Medicine, Southern Medical University, Nanjing, China; ^2^ Department of Neurosurgery, The Affiliated Suqian First People’s Hospital of Nanjing Medical University, Suqian, China; ^3^ Department of Neurosurgery, Jinling Hospital, Medical School of Nanjing University, Nanjing, China; ^4^ Department of Neurosurgery, The 904th Hospital of the Joint Logistics Support Force of Chinese People’s Liberation Army, Wuxi, China

**Keywords:** subarachnoid hemorrhage, astaxanthin, nanoparticle delivery, triggered release, early brain injury

## Abstract

Subarachnoid hemorrhage (SAH) is a fatal disease. Within 72 h of SAH, the intracranial blood-brain barrier (BBB) is destroyed, and the nerve cells have responses such as autophagy, apoptosis, and oxidative stress. Antioxidation is an essential treatment of SAH. Astaxanthin (ATX) induces cells’ antioxidant behaviors by regulating related signal pathways to reduce the damage of brain oxidative stress, inflammation, and apoptosis. Because of its easy degradability and low bioavailability, ATX is mainly encapsulated with stimulus-responsive nanocarriers to improve its stability, making it rapidly release in the brain and efficiently enter the lesion tissue. In this study, the ultrasonic cavitation agent perfluorocarbon (PFH), ATX, and fluorescent dye IR780 were loaded with polydopamine (PDA) to prepare a US triggered release nanoparticles (AUT NPs). The core-shell structure of AUT NPs formed a physical barrier to improve the bioavailability of ATX. AUT NPs have high ATX loading capacity and US responsiveness. The experimental results show that the AUT NPs have high stability in the physiological environment. Both US and pH stimuli can trigger the release. Under US, PFH breaks through the rigid shell. The structure of AUT NPs is destroyed *in situ*, releasing the loaded drugs into neuronal cells to realize the antioxidant and antiapoptotic effects. The *in vivo* experiment results show that the AUT NPs have good biosafety. They release the drugs in the brain under stimuli. The *in vivo* treatment results also show that AUT NPs have an excellent therapeutic effect. This approach presents an experimental basis for the establishment of Innovative SAH treatments.

## 1 Introduction

SAH is one of the most severe stroke emergencies caused by intracranial blood in the subarachnoid space, which is induced by various diseases (Sehba et al., 2012). Early brain injury (EBI) refers to the intracranial lesions occurring within 72 h after SAH and before the formation of cerebral vasospasm. The destruction of the BBB, autophagy of nerve cells, apoptosis, and oxidative stress during EBI are considered to be the leading causes of high disability and mortality in SAH patients (Cahill et al., 2006; Sehba et al., 2012). Among them, the oxidative stress is the primary pathogenesis of cerebral vasospasm and early brain injury after SAH (Zhang et al., 2014a). The common antioxidant stress drugs include resveratrol (Ham and Raju, 2016), glycyrrhizin (Zhang et al., 2014b; Shao et al., 2014) and astaxanthin (ATX) (Farruggia et al., 2018; Wang et al., 2019) etc. Astaxanthin is a natural antioxidant and has the effects of anti-radiation, cardiovascular aging alleviation, Alzheimer’s disease resistance and anti-cancer. Gang Chen et al. reported that the natural ATX could up-regulate corresponding mRNA and protein expression in the Nrf2/ARE pathways to reduce brain oxidative stress (Chen et al., 2011). Xiangsheng Zhang et al. demonstrated that the ATX administration amplifies of the Akt/Bad pathway and reduces the secondary brain injury after SAH (Zhang et al., 2014c). ATX can significantly reduce the expression and activity of MMP-9, alleviating the in brain edema, BBB injury, neurological deficit, and apoptosis 24 h after SAH (Zhang et al., 2015). High-dose ATX treatment can significantly down-regulate the increased the activity of nuclear factor kappa B and the expression of inflammatory cytokines and intercellular adhesion molecule-1 in mRNA transcription and protein synthesis after SAH, producing a neuroprotective effect by inhibiting brain inflammation (Zhang et al., 2014d). Hui Shen et al. found that ATX has a protective effect on free radical injury and ischemia/reperfusion-induced neurodegeneration *in vivo*. The protective mechanisms include inhibiting the ROS, glutamate overflow and apoptosis (Shen et al., 2009). These studies show that the ATX can significantly reduce the oxidative stress response, the inflammation and apoptosis after SAH.

BBB, a unique biological barrier of the central nervous system, dramatically limits the absorption of therapeutic drugs by the brain and its diseased parts (Huile, 2016). Lipophilic drugs are more likely to go through BBB lipid bilayer and to exert their efficacy (Kreuter, 2012; Gao, 2017). The lipophilicity of ATX makes it easier to be absorbed by the BBB. However, ATX’s poor stability and solubility limit its oral intake and routine intravenous administration, hindering its clinical application as a therapeutic drug (Lichota et al., 2010). In addition, ATX is sensitive to light, oxygen and temperature etc. It is prone to be degraded, resulting in decreased biological activity and discoloration (Naguib, 2000). In the recent 10 years, the development of nanoscience has extensively promoted the application of nanomaterials in the biomedical field. Compared with traditional molecular drugs, nano drugs have the characteristics of targeting, slow-release and enhance drug stability (Brightman and Kaya, 2000; Yu et al., 2011; Vries et al., 2016). Zongqi You et al. developed a transferrin-bound polyethylene glycol coated ATX nanoparticles (ATX NPs), which have good water dispersion and biocompatibility (You et al., 2019). Compared with free ATX, ATX NPs containing transferrin show a substantial neuroprotective effect on OxyHb induced neuronal damage. The apoptosis markers levels were significantly reduced. However, the insufficient release of ATX in the brain region limited its further therapeutic efficacy.

Lipophilic astaxanthin can ensure ATX’s absorption in the brain. Therefore, we should try to accumulate enough ATX in the brain area. The structure, shape and performance of stimulus-responsive nanoparticles will change under the external stimulus. Some macro behaviors of nanoparticles can be regulated by adjusting external stimuli, including pH, temperature, magnetic field, light, etc (Fattal et al., 2004; Rodriguezhernandez et al., 2005; Onaca et al., 2010). This kind of intelligent stimulus-responsive nanoparticles has become a research hotspot in biomaterials. Ultrasound (US) imaging plays an essential role in biomedical imaging because of its high security, low cost, portability, and high spatial and temporal resolution (Perera et al., 2015). Other studies have shown that US can promote the penetration of tissue barriers and increase drug absorption (Rapoport, 2004). Research by Ine lentucker et al. showed that US-triggered DOX liposomes mainly released in the US treatment area. Therefore, the tumor cell ablation ability of DOX liposome microbubbles triggered by US was more obvious than that of DOX liposomes (Lentacker et al., 2010). PFH is a phase-changing material, which changes from liquid to gas under US. It is used as an US contrast agent and US responsive carrier material. Xiaotu Ma et al. developed an ultra-stable PFH siliceous nanodroplet (Ma et al., 2020). High-intensity focused US therapy (HIFU) was used to trigger the co-release of oxygen and chemotherapeutic drug DOX and to enhance US imaging simultaneously. Chemotherapy and remission of tumor hypoxia both down-regulated TGF-β1 and significantly inhibited tumor metastasis. Zhang Lu et al. developed a pH-sensitive PFH encapsulated US nanoprobe (Zhang et al., 2018), which is triggered by low-frequency US. The DOX can be quickly released and absorbed by tumor, showing better tumor treatment effect. However, the poor stability and complex packaging process of PFH droplets limited their further application *in vivo*.

PDA, a kind of melanin polymer with excellent biosafety, which is used as a nano-drug carrier material (Hong et al., 2011). Dopamine can undergo an oxidative crosslinking reaction triggered by dissolved oxygen in water, forming a PDA composite thin layer adhered to the surface of materials. In an alkaline environment, electrostatic interaction exists between PDA and cationic polyelectrolyte. PDA and cationic polyelectrolyte deposit alternately on the material surface, realizing the surface modification (Waite, 2008). Because of its photosensitivity, ATX can be easily photodegraded and lose biological activity. PDA has similar light absorption properties to melanin and a vast absorption spectrum. The hydrophobicity of ATX leads to poor stability and solubility, while PDA has good loading performance and stability (Bernsmann et al., 2010). This study designed and established an US triggered nano delivery system that can stably encapsulate ATX. The core is a PFH droplet loading ATX and IR780. The outer layer is a PDA rigid shell. When triggered by US, PFH can change from liquid to gas and release ATX. PDA protects ATX from light. ATX can be released under US after the nanoparticles passing through the BBB, improving the therapeutic effect of ATX. IR780 is used as a tracer to achieve real-time monitoring.

## 2 Materials and Methods

### 2.1 Materials

PFH (98%) was purchased from Jiuding Co., Ltd. Fluorocarbon surfactant (FS-63) was purchased from Guangzhou jieluhua Co., Ltd. Tris was purchased from Sigma Aldrich Co., Ltd. Dopamine hydrochloride (98%) was purchased from Aladdin Biochemical Technology Co., Ltd. Dimethyl sulfoxide (DMSO) was purchased from Tianjin Fuyu Co., Ltd. Copper sulfate pentahydrate (AR) was purchased from Marklin Co., Ltd. Astaxanthin (ATX, 96%) was purchased from Shanghai Yien Co., Ltd. Hydrogen peroxide was purchased from Shanghai Guoyao Co., Ltd. IR780 (≥95%) was purchased from Sigma Aldrich Co., Ltd. The counting kit-8 (CCK-8) kit was purchased from Dojindo Co., Ltd., Japan. HL-7702 human stem cells, IMR-90 human embryonic lung fibroblasts, HEK-293 human embryonic kidney cells, HUVECs human umbilical vein endothelial cells, HT-22 mouse hippocampal neuron cell line, 3T3-L1 mouse embryonic fibroblasts, mouse embryonic fibroblasts were all derived from ATCC. BALB/C mice were purchased from Beijing Huafukang Co., Ltd. Unless otherwise stated, all the chemicals and reagents were of analytical grade and used as received. All animal procedures were approved by the Animal Care and Use Committee of Jinling Hospital and were in accordance with the guidelines of the National Institutes of Health on the care and use of animals.

### 2.2 Preparation of AUT NPs

4 ml Tris solution (0.605 mg/ml, deionized water), 100 µl PFH solution and 200 µl FS-63 solution were fully mixed. A 100% power ultrasonic cleaning machine (KQ-400KDE, Kunshan shumei Co., Ltd.) was used to treat the mixture for 5 min until it changed to a milky white nanoemulsion. 1 ml ATX solution (20 mg/ml, DMSO), 200 µl IR780 solution (3.335 mg/ml, DMSO), 5 ml dopamine hydrochloride solution (10 mg/ml, deionized water) and 150 µl CuSO_4_ solution (1.25 mg/ml, deionized water) and 300 µl H_2_O_2_ solution (0.122 mg/ml) were slowly added to the nanoemulsion. The emulsion was stirred for 5 min and rotated in the dark for 24 h to obtain AUT NPs.

### 2.3 Characterizations of AUT NPs

The particle size and zeta potential distributions of AUT NPs were measured by Malvin nano sizer (ZEN3690, Shanghai Specter Instrument System Co., Ltd.). The morphology of AUT NPs was observed by transmission electron microscope (TEM). AUT NPs were dispersed in normal saline, complete medium, and serum to simulate different physiological environments. The stability was evaluated by particle size changes. ATX was first released by US and acidolysis, and the drug concentration was determined by the HPLC system. The encapsulation rate and drug loading capacity were calculated.

### 2.4 *In vitro* Release and US Tests of AUT NPs

The *in vitro* release test has three groups: general release, pH response release, and US triggered release. The concentration of residual ATX was measured by *in vitro* dialysis method, and the release rate was calculated. The sample was divided into several parts with same volume, and filled into dialysis bags then continuously dialyzed as normal process. In each time point, a dialysis bag was took, and collected the sample. The samples were sufficiently precipitated and centrifuged. The supernatant was collected and then dried by freeze-drying, decomposed by acid liquor. The residual was dissolved by chloroform for HPLC measurement of ATX. The release rate at different time points was calculated. The free ATX was used as the control group.

The purpose of the pH-responsive release is to detect the decomposition performance of nanocarriers at different pH values. The release curves of AUT NPs at different pH values were measured. The three pH values selected to simulate the normal physiological condition and the primary and secondary lysosomes environments were 7.4, 5.2, and 6.5, respectively.

In the *in vitro* US experiments, the US trigger response of AUT NPs was observed by a medical US instrument (voluson E8, Ge, USA). SonoVue, a commercialized US contrast agent, and ultrapure water were used as the control groups.

PDA is black and has a light absorption ability. Therefore, with PDA’s tight encapsulation, the fluorescence intensity of IR780 is low. Once triggered, IR780 will have apparent fluorescence under excitation. Therefore, the US triggered release was evaluated by the fluorescence of IR780. AUT NPs were divided into six groups and put into a black 96-well plate. The samples were triggered by US for 0 s, 60 s, 120 s, 180 s, 240 s, and 300 s, respectively. The US triggering frequency was 1 kHz, the Power amplifier was 16, and the voltage was 9V.

### 2.5 *In vitro* Cytotoxicity of AUT NPs

The *in vitro* cytotoxicity of AUT NPs was detected by CCK-8 assays conducted on HL-7702, IMR-90, HEK-293, HUVECs, HT-22, and 3T3-L1 cell lines. The ratio of ATX to IR780 doses in AUT NPs was approximately 10:1. Therefore, a similar drugs concentration ratio was set for the assay. Each cell line was inoculated on 96-well plates at 1 × 10^4^/ml and incubated with ATX, IR780, AUT NPs, and DMSO for 24 h. After that, the cells in each well were incubated with 10 µl CCK-8 solution for 2 h. The 450 nm absorbance of each well was measured by a microplate reader (GloMax Discover, Promega Corporation) to calculate the cell survival rate.

The cytotoxicity of AUT NPs was further verified by clone formation tests. The cells were evenly dispersed in four 6 cm plates at 700 cells/plate. The normal saline, IR780, ATX, and AUT NPs were added to the plates, respectively. The cells were incubated with the samples until the number of colons in the normal saline group was greater than 50. After that, 1 ml of 4% paraformaldehyde was added to each plate to fix the cells. The plates were then washed with PBS solution. 1ml crystal violet dye was added. The number of colons was recorded.

### 2.6 Cell Internalization Experiment

In order to test the *in vitro* delivery performance of AUT NPs, DOX and coumarin-6 were labeled on AUT NPs to generate two fluorescent signals (Xia et al., 2014; Wang et al., 2018). The primary mouse neuronal cells were inoculated into a confocal special culture plate (1×10^5^ cells/ml). When the cells grew to 40%, the fluorescent-labeled AUT NPs were added and incubated with the cells for different time lengths. The cells were fixed by 4% paraformaldehyde solution, and the nuclei were labeled by DAPI. The cellular internalization of AUT NPs was observed by a laser confocal microscope.

### 2.7 *In vivo* Toxicity of AUT NPs

AUT NPs are injected intravenously into the body. Therefore, it is necessary to evaluate whether AUT NPs and their components can cause hemolysis. The hemolysis test was performed to evaluate DIST NPs’ effect on red blood cells. 4% red blood cell suspension was prepared and divided into groups. The TritonX-100 (0.1%), water, normal saline, solvent (3% DMSO), empty NPs (PFH + FS63 + PDA), PDA, ATX, AUT NPs, and IR780 were added to the test groups, respectively. The blood samples were incubated for 2 h before being centrifuged at 12,000 rpm for 3 min. The 545 nm absorbance of supernatant in each group were measured by a UV-Vis spectrophotometer to calculate the hemolysis rate.

In the acute toxicity test, 70 BALB/c mice, half male and half female, weighing about 20g, were selected and randomly divided into seven groups. Each group has five male and five female mice. The AUT NPs, PDA, FS-63, PFH, DMSO, ATX, and normal saline were intravenously injected into the mice, respectively. The mice were observed continuously for 14 days. The signs were recorded, and the survival rate was calculated. Finally, the surviving mice were euthanized. The main organs were taken for H&E staining and histopathological analysis.

The levels of interleukin-6 (IL-6) and tumor necrosis factor (TNF- α) in the blood of mice were tested by the ELISA kit to evaluate whether DIST NPs can cause a systemic inflammatory reaction *in vivo*. The grouping is the same as the acute toxicity test except that there are four mice in each group. The samples were injected through the tail vein, and the blood samples were collected 24 h after injection.

All experimental protocols in this study were approved by the Animal Care and Use Committee of Southern Medical University and conformed to the Guide for the Care and Use of Laboratory Animals published by the National Institutes of Health.

### 2.8 *In vivo* Triggering of AUT NPs

BALB/C white mice were used to evaluate the *in vivo* triggering effect of AUT NPs. IR780 was used for *in vivo* imaging. The mice were divided into four groups including IR780 + US, high concentration AUT NPs (0.30 mg/ml IR780) + US, low concentration AUT NPs (0.05 mg/ml IR780) + US, and low concentration AUT NPs groups. The sample was injected into the mice intravenously. The US was applied for 60 s for X times, with intervals of 1 h. At the end of each US treatment, the mice images were captured by an IVIS imaging system (excitation wavelength 780 nm, emission wavelength 845 nm). The US parameters were the same as those of the *in vitro* test. After the observation, the mice were euthanized, and the main organs and tumor tissues were taken out to analyze the distribution of AUT NPs *in vivo*.

### 2.9 *In vivo* Therapeutic Effect of AUT NPs

The SAH mice model was prepared by the optic chiasmatic pool blood injection method. A C57BL/6 mouse, approximately 25 g in weight, was fixed under anesthesia. The mouse scalp was disinfected with 75% ethanol solution. We cut slowly along the middle line of the mouse scalp and opened the skin on the front skull. A 0.9 mm drill bit was used to drill a 4.5 mm diameter hole in front of the mouse’s anterior fontanelle (transparent cerebrospinal fluid outflow indicated a successful penetration). And then, 60 µl of the left ventricular artery was extracted. The syringe went from the hole to the bottom of the skull at a 45° angle. 60 µl arterial blood was slowly injected (the same amount of normal saline was injected into the sham group mice). The needle was placed for 5 min to avoid reflux. The hole was sealed by bone wax after the needle was removed. After its scalp was sutured, the mouse was placed on a surgical insulation blanket. The mice were randomly divided into sham, SAH, SAH + empty nanoparticle, SAH + PDA, SAH + ATX, and SAH + ATX NPs treatment groups. The samples were injected intravenously at 24 h post-SAH.

The neurobehavioral scores (Table 1) in each group, including diet, activity, and neurological deficit, were independently completed by professionals(Chen et al., 2011; Zhang et al., 2014a; Zhang et al., 2014c). The specific evaluation criteria are as follows: 1) no neurological deficit (0 points), 2) Suspected neurological deficit (1 point), 3) Mild neurological deficit (2-3 points), 4) Severe neurological deficit (4-6 points).

**TABLE 1:** Behavior and activity scores.

Category	Behavior	Score
Appetite	Finished meal	0
	Left meal unfinished	1
	Scarcely ate	2
Activity	Walked and reached at least three corners of the cage	0
	Walked with some stimulation	1
	Almost always lying down	2
Deficit	No deficits	0
	Unstable walk	1
	Impossible to walk	2

Malondialdehyde (MDA) activity test was performed after anesthesia. The limbs of mice were fixed, and the chest and abdominal cavity were cut to expose the heart. The precooled normal saline was injected into the left ventricle, and the right auricle was cut off. The infusion was continued until the replacement was completed. The brain tissue was quickly removed and stored in an EP tube. RIPA lysate was added. After US lysis and centrifugation, the total protein was extracted and quantified by a BCA protein concentration determination kit. According to the mechanism of the color reaction between MDA and thiobarbituric acid, we detected the 535 nm absorbance of the sample by enzyme labeling instrument. The unit of MDA activity as nmol/mg protein.

Brain water content was measured after anesthesia. The mice were infused with 0.01 mol/L PBS buffer through the left ventricle as described above. After infusion, the mice were euthanized. The brains were taken out and weighed as “wet weight.” The brains were then put into a constant temperature drying oven at 100°C for 72 h. When completely dried, the brains were weighed again as “dry weight.” The water content fraction of brain tissue = (wet weight dry weight)/wet weight×100%。

TUNEL staining was performed after the infusion. The brain specimens were soaked in chloral hydrate and made into paraffin sections. The bilateral temporal lobes were stained by TUNEL staining.

### 2.10 Statistical Analysis

All results are shown as the mean ± SD (standard deviation). SPSS 18.0 (SPSS Inc., Chicago, IL, US) was utilized to perform all statistical analyses. The measurements were subjected to one-way ANOVA. The Mann–Whitney U-test was used to compare the behavior and activity score among groups. A *p* value of <0.05 was considered statistically signifificant.

## 3 Results

### 3.1 Characterization of AUT NPs

**FIGURE 1:**
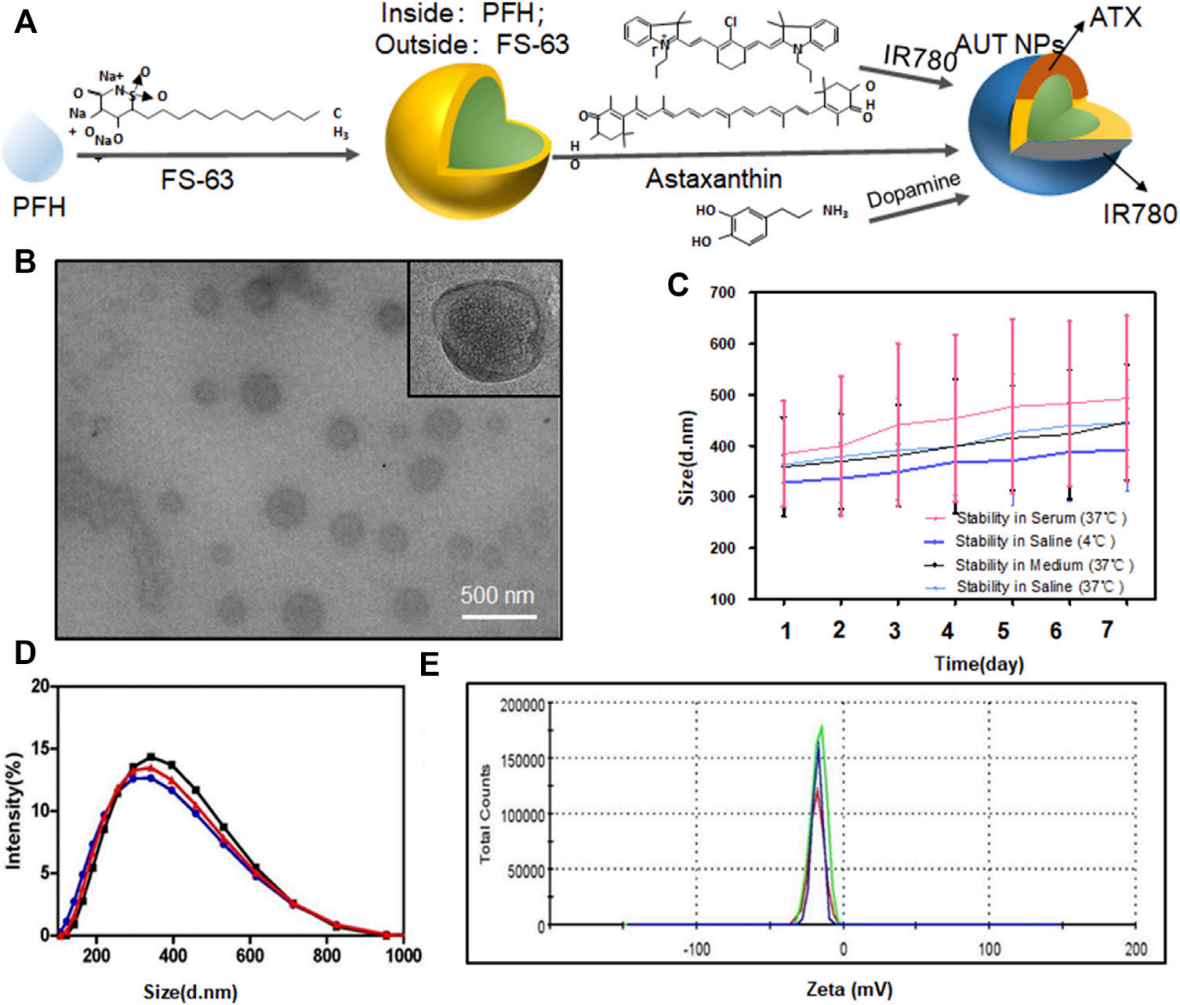
The characterization of AUT NPs. The structural diagram of AUT NPs **(A)**. The TEM results of AUT NPs **(B)**. The stability of AUT NPs under different solvent conditions **(C)**. The size **(D)** and Zeta potential **(E)** distribution of AUT NPs (*n* = 3).

Figure 1A is the structural diagram of AUT NPs. The AUT NPs were characterized, and the morphology and particle size of AUT NPs were observed by transmission electron microscope. As Figure 1B shows, the AUT NPs are spherical and monodisperse. They have apparent hollow and shell structure, consistent with our assumption. Figures 1D,E show that the average particlce size and zeta potential of AUT NPs are 308.4 ± 51.9 nm and - 11.2 ± 2.1 MV, respectively. AUT NPs were dispersed in normal saline, complete medium, and fetal bovine serum, respectively, to simulate different physiological environments. As shown in Figure 1C, the particle size of AUT NPs did not change significantly in 7 days, indicating an excellent colloidal stability of AUT NPs. The ATX encapsulation rate and drug loading capacity of AUT NPs were 94.1 ± 3.1% and 8.7 ± 2.2%, respectively. The IR780 encapsulation rate and drug loading capacity of AUT NPs were 95.8 ± 2.8% and 0.8 ± 0.4%, respectively. These results indicate that the AUT NPs have excellent encapsulating performance.

### 3.2 The Sensitive of Drug Release by Ultrasound Trigger and Under Different pH Conditions *In vitro*

**FIGURE 2:**
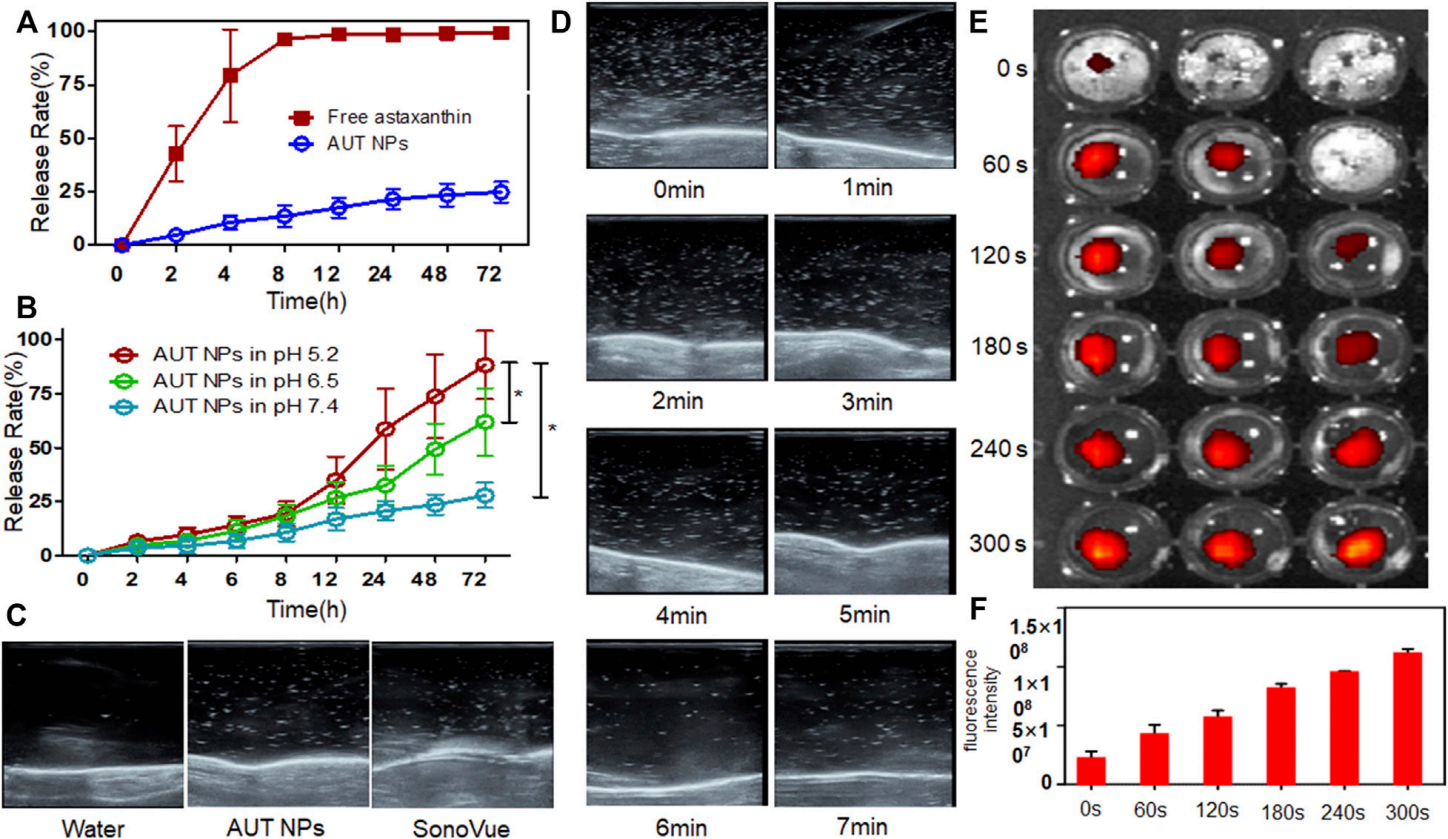
The drug release of AUT NPs under normal **(A)** and pH stimulus **(B)**. The US triggering images with different contrast agents **(C)**. The real-time ultrasonic images of AUT NPs at each time points **(D)**. The IR780 fluorescent images under US **(E)**. The fluorescent intensity changes of IR780 under a series of US triggering time lengths **(F)**. The* represents *p* < 0.05, (*n* = 3).

AUT NPs’ nanoshell collapses under US and the acidic environment *in vitro*, releasing ATX. Therefore, it is crucial to study the nanoparticles’ sensitivity to the stimuli. Here, pH-responsive release and regular release were set as control groups. In the regular release group, ATX was released entirely within 8 h (Figure 2A). In the AUT NPs group, ATX was released slowly. 75% of ATX was still encapsulated in AUT NPs after 72 h, demonstrating an outstanding stability of AUT NPs. When the pH value decreased, the drug release rate of AUT NPs gradually increased (Figure 2B). With a pH of 7.4, the 72 h release rate of ATX was only 25%, indicating that AUT NPs would not release under a typical physiological environment. When pH equaled 6.5, the 72 h release rate of ATX jumped to 60%. With a pH value of 5.2, the release rate of ATX increased rapidly after 12 h, exceeding 75% at 72 h. These results are consistent with the theoretical speculation. After that, the US triggered release of AUT NPs was observed by a clinical US instrument. SonoVue, a commercialized US contrast agent, and water were used as the control groups. The results show that both the AUT NPs and SonoVue generated strong harmonic signals under a certain sound pressure (Figure 2C). The real-time harmonic image monitoring of AUT NPs group at different time points shows that the US microbubbles in AUT NPs solution can be effectively preserved within 4 min. After that the microbubbles gradually disappear and broken (Figure 2D). This result indicates that the US response of AUT NPs is excellent. In the US triggered release experiment (Figure 2E), the fluorescent intensity of the solution gradually increased with the extension of the triggering time, indicating that the IR780 dye was continuously released. Quantitative statistics were made on the fluorescence intensity of IR780 under different triggering stimuli. The results show that the fluorescence intensity of IR780 increased continuously with the extension of triggering time, indicating that prolonging the US triggering time will promote the rupture of AUT NPs’ shell to release the loaded drug (Figure 2F).

### 3.3 *In vitro* Toxicity of AUT NPs

**FIGURE 3:**
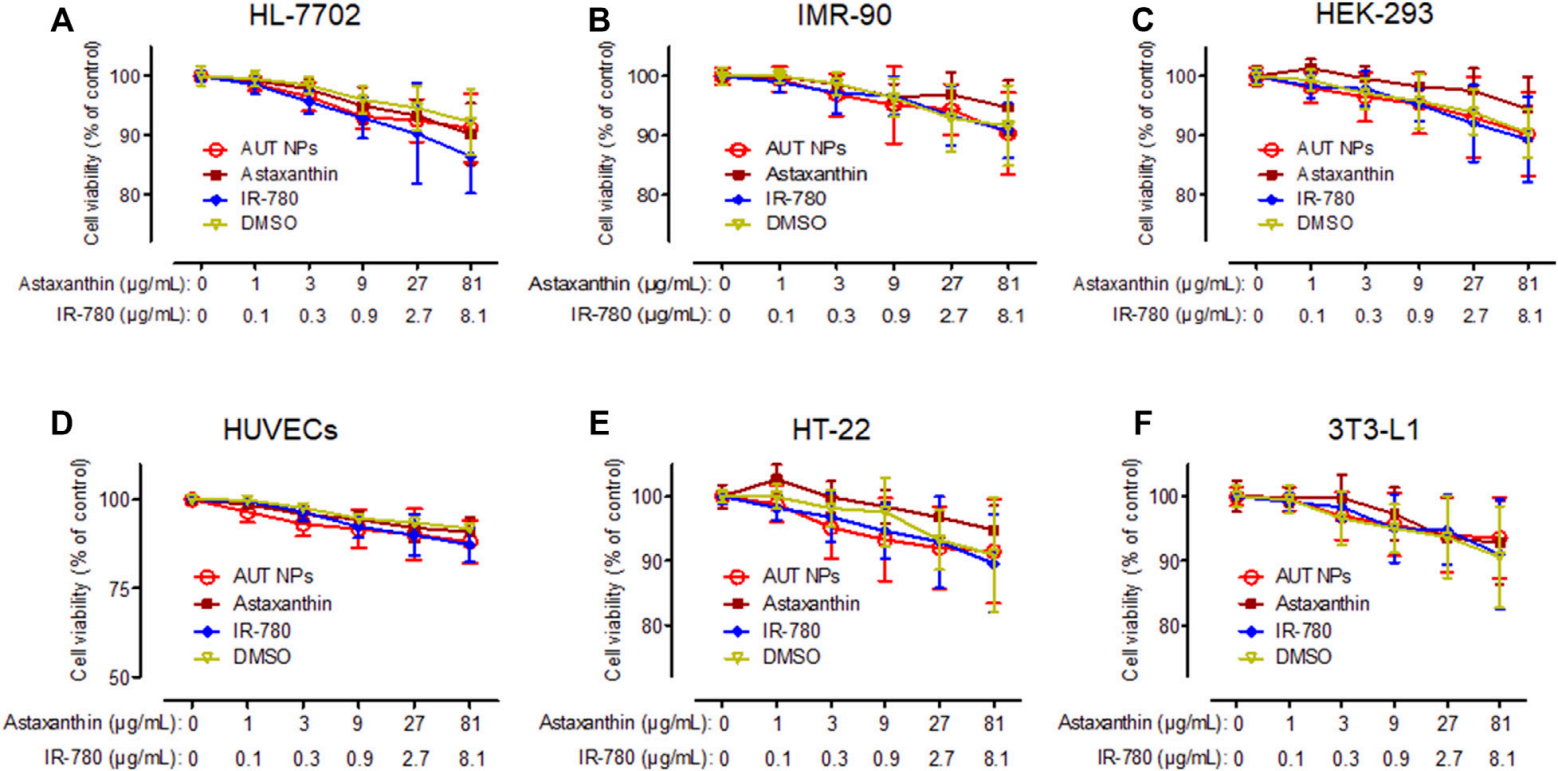
The *in vitro* cytotoxicity results of AUT NPs, ATX, IR780, and DMSO in HL-7702 **(A)**, IMR-90 **(B)**, HEK-293 **(C)**, HUVECs **(D)**, HT-22 **(E)**, and 3T3-L1 **(F)** cells.

Figure 3 shows the CCK-8 test results of AUT NPs, ATX, IR780, and DMSO on different cell lines. We can see from the figure that all the samples do not have apparent cytotoxicity except for the weak cytotoxicity of IR780 at high concentrations. The cell survival rate was above 90%, indicating a low cytotoxicity of AUT NPs.

**FIGURE 4:**
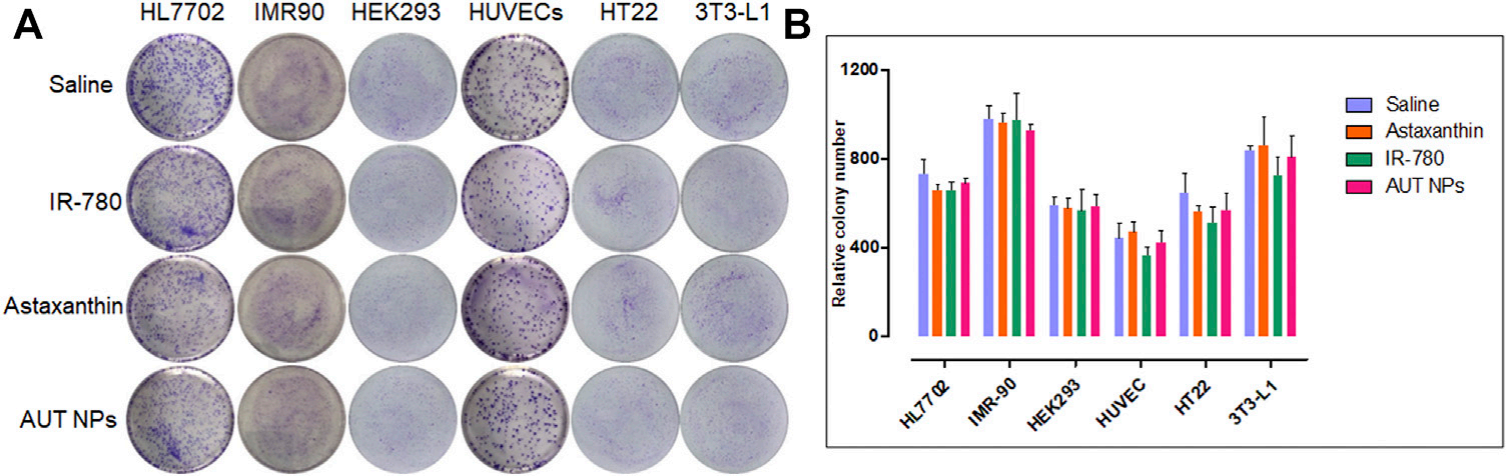
The colony formation assay results of AUT NPs and their ingredients in different cell lines. The colony photographs throughout the assay **(A)** and the quantitative analysis result **(B)**.

In addition to the CCK-8 test, a colony formation assay was conducted to detect the effect of AUT NPs on the proliferation of single cells. As shown in Figure 4, the numbers of colonies in each group are similar except for that of the IR780 treated group, which is less than those in the other groups. These results, together with the CCK-8 test results, confirm the excellent biocompatibility of AUT NPs.

### 3.4 Cell Internalization of AUT NPs

**FIGURE 5:**
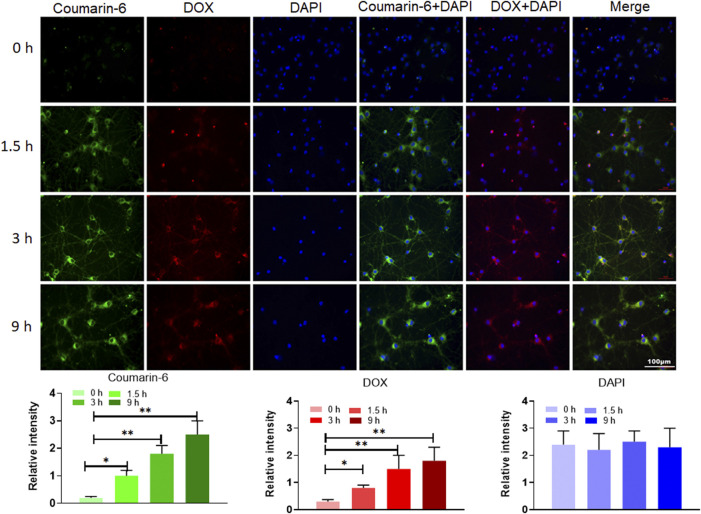
The internalization assay of AUT NPs. Photographs in top are fluorescent images of cell in different time points. Histograms in bottom are quantitative analysis of fluorescent intensities in different time points. The **p < 0.01; The *p < 0.05.

To evaluate the cell internalization of AUT NPs, we prepared the coumarin-6 and DOX dual fluorescent-labeled AUT NPs. As Figure 5 shows, the fluorescence of coumarin-6 and DOX were mainly accumulated in the cytoplasm. The merged image of coumarin-6, DOX, and DAPI further prove this scenario. With the extension of time, the signals of coumarin-6 and DOX gradually increased in the cytoplasm. The fluorescent intensity quantitative results show that, compared with the DAPI signals in the nuclei, the fluorescent intensity of coumarin-6 and DOX has a gradient in continuous time. These results indicate that AUT NPs can effectively enter cells and release drugs in the cytoplasm.

### 3.5 *In vivo* Toxicity of AUT NPs

**FIGURE 6:**
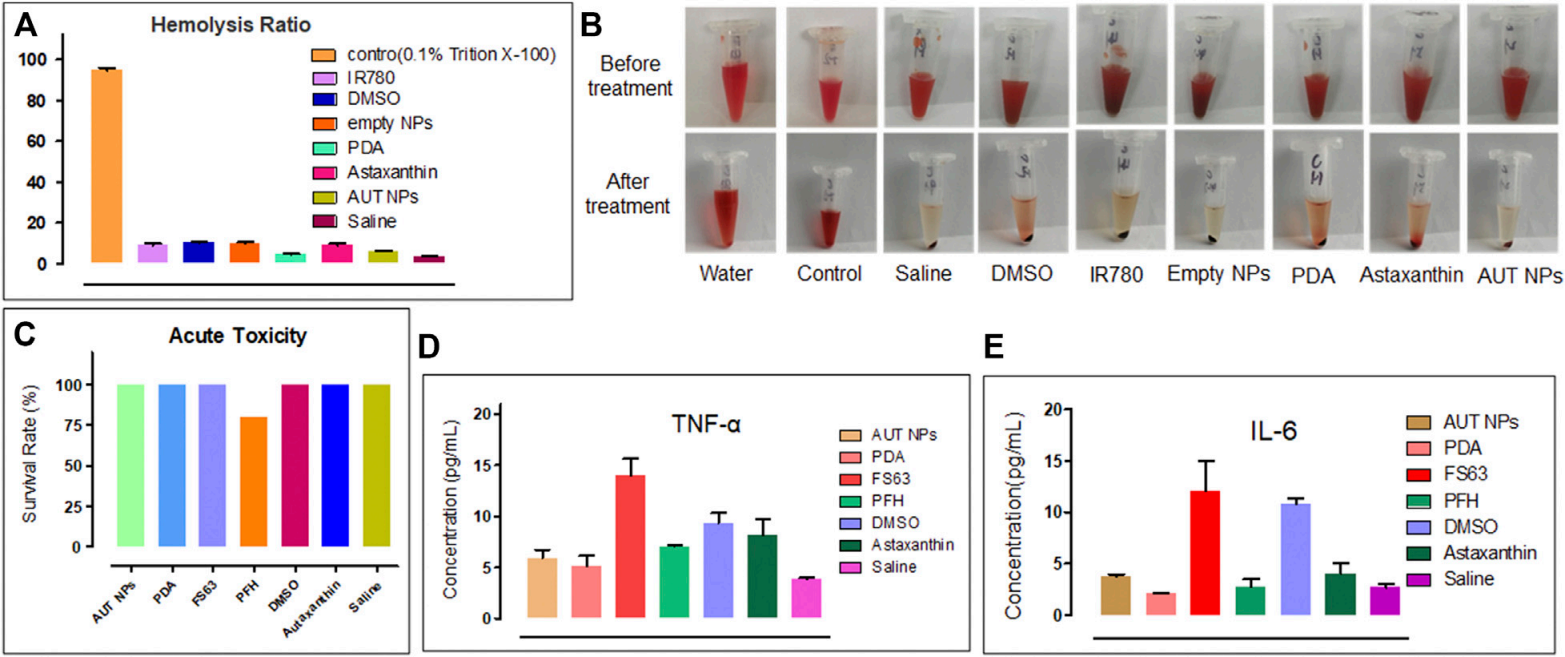
*In vivo* toxicity test of AUT NPs. The results **(A)** and photographs **(B)** of the hemolysis experiment. The results of acute toxicity test of AUT NPs **(C)**. The TNF- α **(D)** and IL-6 **(E)** inflammatory markers detection results of AUT NPs and the other control materials.

**FIGURE 7:**
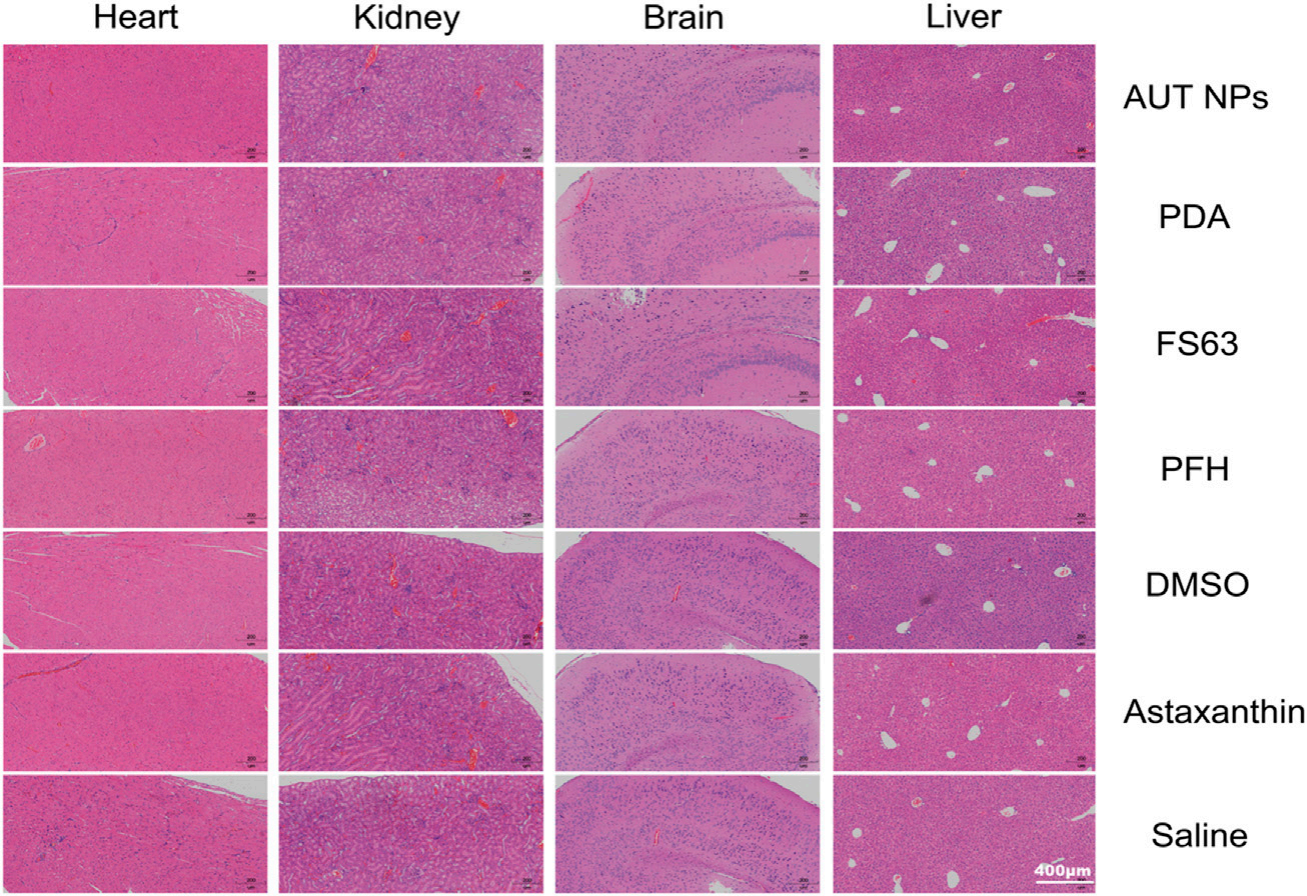
The organs histopathological assay of AUT NPs in acute toxicity.

To evaluate the *in vivo* safety of AUT NPs, the hemolysis rate of AUT NPs was detected. The results show that the hemolysis rate of AUT NPs was low and only slightly higher than that of PDA and normal saline (Figure 6A). After treatment and centrifugation, many erythrocyte precipitations can be seen in the AUT NPs group’s tube. No apparent hemolysis was found in the other groups (Figure 6B). In an acute toxicity experiment, we also found that two mice were died shortly in PFH treated group This scenario may be due to PFH gasification, leading to the cardiac embolism. The survival rate of mice was 100% (Figure 6C) also indicated that AUT NPs conclude its ingredients with fine biosafety and will not cause noticeable toxic and side effects. Finally, to evaluated whether AUT NPs provoke severe systemic inflammatory response was proposed detection by inflammatory factors tests. The results show that the concentration of TNF- α was lower than 10 pg/ml, which is close to the PDA and normal saline (Figure 6D). The concentration of IL-6 was similar to that of normal saline with lower than 5 pg/ml (Figure 6E). It predicted that AUT NPs would not raise the concentration of TNF- α and IL-6 *in vivo*. Thus, avoid inflammation Effectively. acute toxicity experiment was finished, euthanized mice Subsequently. The heart, liver, kidney, and brain of mice in each group were collected for H&E staining and histopathological analysis. The results show that the nucleus structure of brain cells was complete, and no noticeable histopathological changes were found (Figure 7). In general, AUT NPs have no apparent tissue and cell damage and will not cause tissue necrosis.

### 3.6 The *in vivo* Distribution of AUT NPs

**FIGURE 8:**
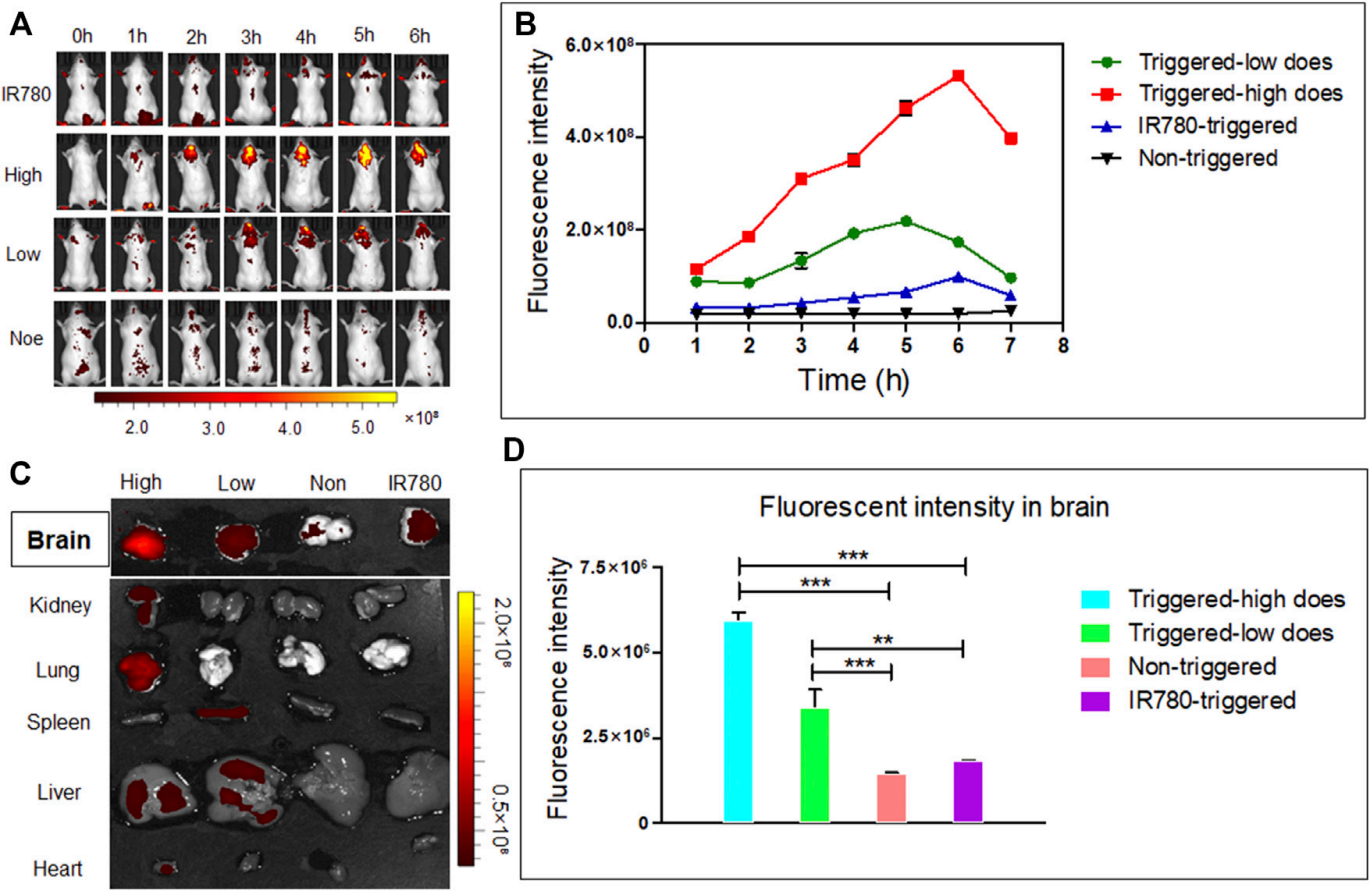
*In vivo* distribution and brain release of AUT NPs in mice: *in vivo* fluorescence images **(A)**, *in vivo* fluorescence quantitative analysis of brain contact sites **(B)**, fluorescence images of the brain and other primary organs **(C)**, quantitative fluorescence analysis of the brain **(D)**. The **p < 0.01; and ***p < 0.001.

The mice were divided into four groups. In the high concentration AUT NPs treated group, the fluorescent intensity of the mice brains was significantly enhanced 2 hours after injection under US triggering. With the increase of trigger times, the brain signal was also enhanced and reached its highest intensity at 6 h, indicating that the US cracked the shell of AUT NPs and triggered the release of IR780 and other molecules. In the low concentration AUT NPs treated group and the mice brains also had stronger fluorescent signals than those of the other parts of the body. Compared with the free IR780 treated group, the signals in the two AUT NPs groups were mainly concentrated in brain triggering sites. In the non-triggered group, no significant signal was found in the brain until 6 h. Thus, US induces the collapse of AUT NPs *in vivo*, releasing endogenous substances at the triggering sites (Figure 8A). The results show that the fluorescent signal intensity of the AUT NPs group in the US triggered group was much higher than those of the non-triggered group and the free IR780 group. With the extension of triggering time length, the signal of US triggered groups gradually enhanced, while the signal of non-triggered group remained unchanged. The signal of the free IR780 group was not affected by US. The enhancement of signal at 6 h may be due to the accumulation of IR780 in the systemic circulation. US has a penetration effect on BBB. In general, the release of IR780 was positively related to its concentration and US triggering time length (Figure 8B). The brain and organs fluorescent analysis of mice after dissection shows that AUT NPs mainly accumulated in the liver and lung due to the structural characteristics of nanoparticles. The brain signals were extracted separately. We found that the AUT NPs released IR780 after being triggered through the skull tops of the mice. The higher the concentration, the more IR780 released (Figure 8C). Quantitative analysis of the extracted brain tissue shows that the signal of AUT NPs in the triggered group was more robust than those in the non-triggered group and control group, indicating that the AUT NPs have a good US response and release performance.

### 3.7 *In vivo* Treatment of AUT NPs

**FIGURE 9:**
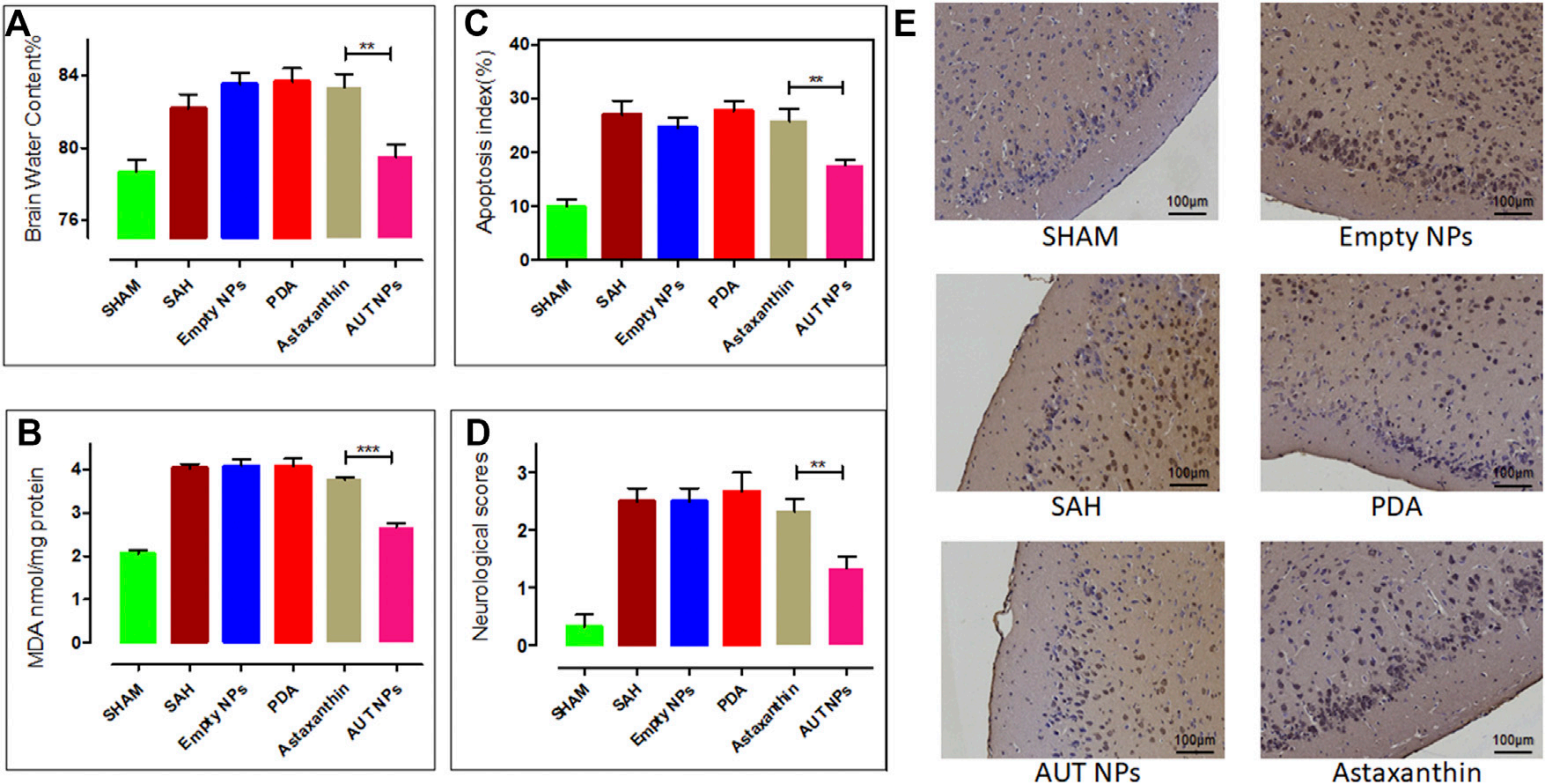
The *in vivo* therapeutic effect of AUT NPs, including the brain water content **(A)**, the brain MDA level **(B)**, the statistics of apoptosis rate **(C)**, the neurological score **(D)**, and the TUNEL stained slice images of brain tissues in different groups **(E)**. The **p < 0.01, (*n* = 6); and ***p < 0.001, (*n* = 6).

Next, after 48 h of AUT NPs injection, a series of studies were carried out to evaluate the therapeutic effect. According to the neurobehavioral scores of mice in each group, the SAH model mice in the AUT NPs treatment group have the lowest score of 1-2, which is much lower than SAH model mice in other groups (Figure 9A). As Figure 9B shows, the water content of SAH model mice in the AUT NPs treatment group was similar to that of the healthy mice and significantly lower than those in ATX, PDA and empty nanoparticle treated groups. These results indicate that mice’s brain biochemical tissue composition and metabolic activity in the AUT NPs treatment group were similar to healthy mice, while the other experimental groups were abnormally high. MDA content is an important indicator reflecting the potential antioxidant capacity of the body. It reflects the rate of lipid peroxidation and the degree of tissue peroxidation damage. The results in Figure 9C show that the MDA concentration of AUT NPs treated mice was close to that of healthy mice and were much lower than that of the control group mice, indicating that the AUT NPs can reduce the tissue lipid oxidation levels. Finally, the apoptosis data of brain cells were collected, and the apoptosis index was calculated. The results show that the apoptosis index of the AUT NPs treated mice was significantly lower than those of the ATX, PDA, and empty nanoparticle treated groups and was slightly higher than that of the healthy mice. The AUT NPs could significantly reduce the apoptosis rate and prevent the additional damage of brain cells. The images of TUNEL staining also confirmed this phenomenon to detect bilateral temporal lobe cell apoptosis (Figure 9E).

## 4 Discussion

Mainly caused by aneurysm rupture, SAH, a disease with a mortality of up to 40% (Dority and Oldham, 2016), is usually accompanied by a series of adverse reactions such as local inflammatory cascade, systemic inflammatory response, apoptosis, endothelial cell dysfunction, and oxidative stress (Ayer and Zhang, 2010). As a multi-target neuroprotective agent, ATX participates in neuroprotection through anti-inflammatory, antioxidant, and anti-apoptosis mechanisms. However, ATX’s application is limited because it has poor water solubility and could be easily absorbed by intestinal cells and degraded by light, oxygen stress, and thermal stress (Baranska and Kaczor, 2015). Nanomedicine improves the pharmacokinetics, administration, and bioavailability of drugs through biodegradable nano aggregates. At present, there are many kinds of nano delivery systems (Gautam et al., 2021), including ATX lipid carrier (LBC) and ATX polymer system, to enhance the stability, hydrophilicity, and antioxidant capacity of ATX (Tamjidi et al., 2018). However, in the treatment of central nervous system diseases, the existence of BBB hinders the delivery of most drugs to the brain. In recent years, microbubbles and US have shown great potential in penetrating temporal BBB. Through cavitation, microbubble enables the non-invasive stimulating drugs to be transmitted to the brain. It realizes the passive diffusion of drugs and significantly enhances the accumulation of drug molecules in the brain (Hynynen, 2008; Sheikov et al., 2008; Armulik et al., 2010; O’Brown et al., 2018). This study found that ATX and PFH can be loaded into PDA through a non-covalent bond to form a spherical nanoparticle with a stable structure. The dispersion and stability of AUT NPs also play a key role in their biological applications (Curley et al., 2017). In DLS measurement, the size distribution of AUT NPs did not change significantly within 7 days, indicating good stability of AUT NPs *in vivo*.

There are many reports on the use of PDA to construct stimulus-responsive intelligent materials. However, because of the BBB, those reports are mainly in biological imaging and photothermal transformation and only a few in brain drug response delivery. Cavitation microbubbles can affect the integrity of biofilm according to acoustic pressure, creating acoustic pores on the cell membrane to further stimulate the endocytosis of drugs (Deprez et al., 2021). In this study, we designed three groups of release experiments to detect the responsive release performance of AUT NPs. In the neutral pH environment, the release of ATX in AUT NPs was minor. The release of ATX increased significantly under the simulated lysosomal intracellular environment with US triggering *in vitro*, indicating that a stable release system has been preliminarily constructed. The excessive lysosomes activated in injured neurons after SAH can further stimulate the drug release from AUT NPs. The *in vivo* US response of AUT NPs was detected by fluorescent tracer IR780. Compared with the other parts of the body, the fluorescent signal in the brain was stronger, maintaining for 6 h. The signal intensity in the AUT NPs treated group was positively correlated with the injection dose. In the AUT NPs treated group without US triggering, the intracranial signal was weak. The signal intensity of the main organs shows that the signal was concentrated in the brain. These results show that the AUT NPs have a good US response and release characteristics, in line with our initial design.

Several indicators were used to evaluate the *in vivo* safety, antioxidant effect, and anti-apoptotic effect of AUT NPs on neuronal cells. Through the toxicity test of AUT NPs with multiple cell lines, we found that the AUT NPs have good biocompatibility and could be successfully endocytosed into cells. After SAH, infiltrated neutrophils can release inflammatory cytokines IL-6 and TNF-a, resulting in direct damage to peripheral nerve cells, amplifying leukocyte recruitment and further aggravating EBI (Henriksen-Lacey et al., 2017). The *in vivo* toxicity test further shows that AUT NPs themselves will not cause inflammation and hemolysis and can be used for injection. To evaluate whether AUT NPs have a neuroprotective effect, we need to detect neurological function and brain edema. The results show that AUT NPs can effectively improve the behavior and alleviate brain edema of SAH rats. TUNEL staining images show that healthy mice had sufficient neurons, which usually aligned and have a large nucleus and abundant cytoplasm. The volume and number of neurons in SAH mice decreased. There were a lot of cell fragments, deep cytoplasmic staining, and decreased or disappeared nucleosomes. The AUT NPs could weaken these pathological changes and significantly reduce SAH-induced neuronal apoptosis. MDA, a decomposition product of peroxide polyunsaturated fatty acids in membrane lipids, is considered one of the most sensitive biomolecules to reactive oxygen species for a long time (Cheeseman et al., 1988; Ece et al., 2010). In the experiment, AUT NPs significantly reduced the concentration of MDA. SAH can cause apoptosis of smooth muscle, endothelial cells, neurons, and glial cells. Neuronal apoptosis plays an essential role in SAH pathology (Sabri et al., 2008). The test results of neuronal apoptosis show that AUT NPs can reduce neuronal apoptosis caused by SAH. The apoptosis rate was lower than those of all control groups. The anti-apoptosis effect of AUT NPs was better than that of ATX alone. All the results show that the combination of AUT NPs and US stimulus can inhibit the apoptosis of neuronal cells and the oxidation of cell molecules. Compared with ATX and the other control materials, the final therapeutic effect of AUT NPs is similar to that of healthy rats, showing the best therapeutic effect.

## 5 Conclusion

In this study, AUT NPs were prepared for craniocerebral delivery of ATX. The *in vitro* characterization and release test proved that the AUT NPs have good stability and can release ATX under US and pH stimulation, but ATX will not be released in advance under a non-triggered environment. Therefore, the synthesized AUT NPs meet our initial design expectation. The results of cytotoxicity, hemolysis, and inflammatory factor tests show that AUT NPs have good biosafety and will not cause severe toxic and side effects. *In vivo* triggering experiments show that AUT NPs could achieve stable and long-term release in intracranial. The therapeutic tests of AUT NPs showed that the therapeutic effect of AUT NPs was better than that of free ATX. In general, the treatment approach realizes the combination of clinical ultrasound and drug therapy, it also provides a valuable reference applied to the therapy of brain neuropathy.

## Data Availability

The original contributions presented in the study are included in the article/supplementary material, further inquiries can be directed to the corresponding author.
